# Clinicopathological features and prognostic validity of WHO grading classification of SI-NENs

**DOI:** 10.1186/s12885-017-3490-3

**Published:** 2017-08-04

**Authors:** Luohai Chen, Lin Zhou, Meng Zhang, Liang Shang, Panpan Zhang, Wei Wang, Cheng Fang, Jingnan Li, Tianming Xu, Huangying Tan, Pan Zhang, Meng Qiu, Xianjun Yu, Kaizhou Jin, Ye Chen, Huishan Chen, Rong Lin, Qin Zhang, Lin Shen, Minhu Chen, Jie Li, Leping Li, Jie Chen

**Affiliations:** 1 Department of Gastroenterology, The First Affiliated Hospital, Sun Yat-sen University, No.58 Zhongshan II Road, Yuexiu District, Guangzhou, 510080 China; 2 Department of Gastroenterology, The First Affiliated Hospital of Zhengzhou University, Zhengzhou, China; 3 Department of Gastrointestinal Surgery, Shandong Provincial Hospital Affiliated to Shandong University, No.324 Jingwu Road, Huaiyin District, Jinan, 250021 China; 4 Department of Gastrointestinal Oncology, Key Laboratory of Carcinogenesis & Translational Research under Ministry of Education, Peking University Cancer Hospital & Beijing Cancer Hospital, No.52 Fucheng Road, Haidian District, Beijing, 100142 China; 5 Department of Gastric Surgery, Sun Yat-sen University Cancer Center; State Key Laboratory of Oncology in South China; Collaborative Innovation Center for Cancer Medicine, Guangzhou, China; 6 Department of Gastroenterology, Peking Union Medical College Hospital, Beijing, China; 7 Department of Integrative Oncology, China-Japan Friendship Hospital, Beijing, China; 8 Department of Medical Oncology, Cancer Center, State Key Laboratory of Biotherapy, West China Hospital, West China Medical School, Sichuan University, Chengdu, China; 9 Department of Pancreatic Surgery, Fudan University Shanghai Cancer Center, Shanghai, China; 10 Department of Gastroenterology, Nanfang Hospital, Southern Medical University, Guangzhou, China; 11 Department of Gastroenterology, Union Hospital, Tongji Medical College, Huazhong University of Science and Technology, Wuhan, 430022 China; 12 Department of Pathology, Union Hospital, Tongji Medical College, Huazhong University of Science and Technology, Wuhan, China

**Keywords:** Neuroendocrine neoplasms, Small intestine, Clinicopathological characteristics, Tumor grade

## Abstract

**Background:**

The clinicopathological characteristics of small intestinal neuroendocrine neoplasms (SI-NENs) and the prognostic validity of WHO grading classification for SI-NENs are still unknown in Asian patients.

**Methods:**

277 patients and 8315 patients with SI-NENs were retrieved respectively from eleven Chinese hospitals and Surveillance, Epidemiology, and End Results (SEER) cancer registry. Overall survival was used as the major study outcome. Survival analysis using Kaplan-Meier analysis with log-rank test and cox regression analysis were applied.

**Results:**

Clinicopathological characteristics of SI-NENs were quite different among different races. Duodenum was the predominant tumor site in Chinese patients and Asian/Pacific Islander patients but not in white patients from SEER database. Patients with duodenal NENs tended to have more localized disease than patients with jejunal/ileal NENs which were confirmed by patients from SEER database. Grade 3 or poorly differentiated/undifferentiated tumor were more common and tumor size was significantly larger in ampullary NENs compared with that in non-ampullary duodenal NENs. As for the prognostic validity of WHO grading classification, survival between patients with grade 1 and grade 2 disease was not significantly different. Ki-67 index of 5% might be a better threshold between grade 1 and grade 2 than Ki-67 index of 2% in SI-NENs.

**Conclusions:**

Our study revealed that the clinicopathological characteristics of SI-NENs among different races were quite different. This might because duodenal NENs was much more common in Chinese patients and Asian/Pacific Islander patients. Duodenal NENs and jejunal/ileal NENs, ampullary and non-ampullary duodenal NENs shared different characteristics. Ki-67 index of 5% might be a better threshold between grade 1 and grade 2 in SI-NENs.

## Background

Small intestinal neuroendocrine neoplasms (SI-NENs) is a rare group of malignancies originating from duodenum, jejunum and ileum. Epidemiologic studies from United States and European countries indicate that the incidence of NENs has been rising significantly while small intestine is the most common location of digestive NENs which accounts for 30%–41% of digestive NENs with an age-standardized incidence rate of 0.86/100,000 [1, 2]. However, unlike those in western population, SI-NENs is much rarer in Asian population. An epidemiologic study from Taiwan showed that SI-NENs accounted for 9% of digestive NENs with an age-standardized incidence rate of 0.06/100,000 [3]. Our former single center study revealed that in 178 patients with digestive NENs, only 17 patients (9.5%) had disease located in small intestine [4]. Another nation-wide epidemiologic study of China showed that SI-NENs consisted of only 5.6% of gastroenteropancreatic NENs [5]. Because of the low incidence rate of SI-NENs, clinicopathological characteristics of SI-NENs are still unknown in Asian population.

The current widely used pathological classification for digestive NENs was firstly proposed by European Neuroendocrine Tumor Society (ENETS) and endorsed by World Health Organization (WHO) in 2010 [6, 7]. This WHO grading classification distinguishes NENs into three grades (grade 1, grade 2 and grade 3) according to tumor differentiation, Ki-67 index and mitotic count. Studies from western countries indicated that patients with grade 3 disease had worse outcome compared with patients with grade 1/2 disease [8, 9]. Nevertheless, Ki-67 index of 2% as the threshold differentiating grade 1 and grade 2 disease is challenged by a number of studies. In pancreatic NENs, studies suggested that Ki-67 index of 5% was a better threshold than 2% between grade 1 and grade 2 to predict survival of patients [10, 11]. Study from Khan, et al. also suggested that the thereshold to classify grade 1 and grade 2 should be revised from 2% to 5% both in pancreatic and midgut NENs including NENs of lower jejunum, ileum and appendix [12]. Since Ki-67 index threshold to differentiate grade 1 and grade 2 remained controversial, and there are few studies investigating the prognostic validity of WHO grading classification in the whole small intestine including duodenum jejunum and ileum in Asian patients, whether this grading criteria is appropriate for outcome prediction in SI-NENs of Asian patients is still unclear.

The goals of our study are to investigate the clinicopathological characteristics of Chinese patients with SI-NENs by comparing with patients from Surveillance, Epidemiology, and End Results (SEER) cancer registry, and to investigate the prognostic validity of the WHO grading classification for SI-NENs using a multicenter cohort from China.

## Methods

### Patients and data collection

Clinical data of patients with pathologically confirmed SI-NENs from January 2000 to July 2016 was retrieved from eleven Chinese hospitals including The First Affiliated Hospital, Sun Yat-sen University (*n* = 49), The First Affiliated Hospital of Zhengzhou University (*n* = 36), Shandong Provincial Hospital Affiliated to Shandong University (*n* = 34), Peking University Cancer Hospital (*n* = 31), Sun Yat-sen University Cancer Center (*n* = 26), Peking Union Medical College Hospital (*n* = 25), China-Japan Friendship Hospital (*n* = 24), West China Hospital of Sichuan University (*n* = 17), Fudan University Shanghai Cancer Center (*n* = 12), Nanfang Hospital, Southern Medical University (*n* = 12), Union Hospital, Tongji Medical College, Huazhong University of Science and Technology (*n* = 11). These eleven hospitals respectively located in the north, central, west, east and south of China and all of these hospitals were representative general hospitals or cancer centers in their regions. NENs grown in ampullary region were also included in this study as a part of duodenal NENs. Patients who had previous or concomitant other kinds of cancer or documented hereditary syndromes such as multiple endocrine neoplasia type 1 (MEN-1) were excluded.

We also retrieved data of patients with SI-NENs from the SEER database. We selected all NENs of the small intestine (site code: C17.0 to C17.9) and ampulla of Vater (site code: C24.1) from the SEER database. The following ICD-O-3 histology codes were applied to identify SI-NENs including: 8013, 8150–8156, 8240–8249. Only patients diagnosed with positive pathology after 2000 were included in this study. Patients with a history of other cancers or diagnosed at autopsy or on death certificate were excluded.

Data including age at diagnosis, sex, date of initial diagnosis, location of primary tumor, tumor differentiation, tumor size and extension, nodal status, location of distant metastasis, follow-up data and surgery of primary tumor were retrieved from both the Chinese cohort and SEER database. Surgery of primary tumor included endoscopic resection, local excision, total resection, debulking surgery, et al. Other information including presenting symptoms, tumor grade according to WHO 2010 classification based on Ki-67 index and mitotic count were also retrieved from the Chinese cohort. In SEER database, tumor grade according to WHO 2010 classification was not available, only tumor differentiation was retrieved. All data were reviewed and checked independently by Luohai Chen and Jie Chen and this study was approved by the institutional review board of the included hospitals.

### Grading and staging classification

Ki-67 index and mitotic count were used for assignment of tumor grade in Chinese patients. Ki-67 index was detected using MIB-1 antibody and counted in areas of strongest nuclear labelling. Mitotic count was evaluated at least 50 HPFs (1HPF = 2 mm^2^). Higher grade was assigned when discrepancy between Ki-67 index and mitotic count to determine grade existed. Three grades were classified according to the WHO 2010 classification including: Grade 1 (G1, Ki-67 index ≤ 2% and/or mitotic count<2/10HPF), Grade 2 (G2, Ki-67 index: 3–20% and/or mitotic count: 2–20/10HPF), Grade 3 (G3, Ki-67 index>20% and/or mitotic count>20/10HPF) [13]. All pathological sections were reviewed by specialized expert pathologists from the included hospitals. Tumor stages were assigned according to the staging classification sequentially proposed by European Neuroendocrine Tumor Society (ENETS) and American Joint Committee on Cancer (AJCC) [7, 14, 15] which were identical in NENs of small intestine.

### Statistical analysis

To investigate the basic clinicopathological characteristics of the study patients, student t test, *χ* ^*2*^ test (or Fisher exact test) and Mann-Whitney method were used. Survival time was measured from date of initial diagnosis until the date of death or last follow-up. Overall survival (OS) analyses were then performed using Kaplan-Meier analyses with log-rank test. Multivariate analyses were performed using Cox proportional hazards regression with the lowest risk group as the reference group. Hazard ratios (HRs) and 95% confidence intervals (CIs) were calculated. All analyses were carried out by using IBM SPSS statistics 22.0 (IBM, Chicago, IL), while statistical significance was defined as a 2-sided *P* < 0.05.

## Results

### Comparison of the clinical characteristics of SI-NENs in different races

In total, 277 patients and 8315 patients with SI-NENs were included respectively from the Chinese cohort and SEER database (Table 1). The clinical characteristics of SI-NENs among different races were different. The mean ages were 54.4, 62.4, 60.3 and 60.7 respectively in Chinese, white, black patients and Asian/Pacific Islander (AP) patients. Except for black patients, male patients were more common. Duodenum was the predominant primary tumor site of SI-NENs in Chinese (76.5%) and AP patients (71.9%). But in white patients, jejunal/ileal NENs was more common (68.1%). In Chinese patients, tumor size was significantly larger than that in other groups of patients. Compared with white patients (28.2%), stage I/II were common in Chinese patients (49.8%), black patients (39.3%) and AP patients (47.2%). Surgery of primary tumor was performed in most of the patients in different race patients.

**Table 1:** Comparison of the clinical characteristics of SI-NENs among different races

Characteristics	Chinese Patients (*N* = 277)	SEER database			*P* value
		White patients (*N* = 6711)	Black patients (*N* = 1387)	Asian/Pacific Islander Patients (*N* = 217)	
Age, years					<0.001
Mean(95%CI)	54.4 (52.8–56.0)	62.4 (62.0–62.7)	60.3 (59.6–61.0)	60.7 (59.0–62.5)	
Range	17–83	2–98	21–96	27–95	
Sex					<0.001
Male	157 (56.7%)	3448 (51.4%)	635 (45.8%)	116 (53.5%)	
Female	120 (43.3%)	3263 (48.6%)	752 (54.2%)	101 (46.5%)	
Location of primary tumor^a^					<0.001
Duodenum	212^b^ (76.5%)	1541 (31.9%)	569 (57.5%)	123 (71.9%)	
ampulla	72 (37.1%)	117 (7.6%)	24 (4.2%)	9 (7.3%)	
Non-ampulla	122 (62.9%)	1424 (92.4%)	545 (95.8%)	114 (92.7%)	
Jejunum/ileum	65 (23.5%)	3283 (68.1%)	420 (42.5%)	48 (28.1%)	
Presenting Symptoms					-
Abdominal pain	152 (54.9%)				
Nausea/vomiting	48 (17.3%)				
Bloating	42 (15.2%)				
Jaundice	37 (13.4%)				
GI bleeding	34 (12.3%)				
Diarrhea	27 (9.7%)				
Intestinal obstruction	24 (8.7%)				
Flushing	9 (3.2%)				
Incidental diagnosis					-
Yes	35 (12.6%)				
No (symptomatic)	242 (87.4%)				
Tumor size^c^					<0.001
Median (cm)	2.5	1.6	1.5	1.5	
Tumor grade^d^					-
Grade 1	91 (36.5%)				
Grade 2	81 (32.5%)				
Grade 3	77 (30.9%)				
Tumor differentiation^e^					0.095^f^
Well differentiated		2324 (76.6%)	474 (81.3%)	69 (74.2%)	
Moderately differentiated		553 (18.2%)	84 (14.4%)	15 (16.1%)	
Poorly differentiated/undifferentiated		157 (5.2%)	25 (4.3%)	9 (9.7%)	
Tumor stage^g^					<0.001^h^
I	32 (13.3%)	626 (13.2%)	162 (18.3%)	43 (25.4%)	
II	88 (36.5%)	709 (15.0%)	186 (21.0%)	28 (16.6%)	
III	61 (25.3%)	2077 (43.8%)	331 (37.4%)	59 (34.9%)	
IV	60 (24.9%)	1329 (28.0%)	205 (23.2%)	39 (23.1%)	
Location of distant metastases^i^					-
No	217 (78.3%)				
Liver	52 (86.7%)				
Bone	3 (5.0%)				
Lung	1 (1.7%)				
Brain	1 (1.7%)				
Others	13 (21.7%)				
Surgery of primary tumor					-
Performed	233 (84.1%)	5787 (86.2%)	1097 (79.1%)	166 (76.5%)	
Unperformed or unknown	44 (15.9%)	924 (13.8%)	290 (20.9%)	51 (23.5%)	

*GI* gastrointestinal. ^a^The specific location of 1887 white patients, 398 black patients and 46 Asian/Pacific Islander patients from SEER database were not available. ^b^In Chinese patients, the specific location of 18 patients with duodenal NENs was unknown. ^c^Tumor size was specified in 222 Chinese patients, 5249 white patients, 1008 black patients and 151 Asian/Pacific Islander patients from SEER database. ^d^Tumor grade based on WHO grading criteria was specified in 249 Chinese patients. ^e^In SEER database series, tumor differentiation was available in 3034 white patients, 583 black patients and 93 Asian/Pacific Islander patients. ^f^Well/moderately differentiated and poorly differentiated/undifferentiated were compared among different races. ^g^Tumor stage was specified in 241 Chinese patients, 4741 white patients, 884 black patients and 169 Asian/Pacific Islander patients from SEER database. ^h^Stage I/II and stage III/IV were compared among different races. ^i^Information of location of distant metastases was available in all Chinese patients

In Chinese patients, the most common presenting symptom was abdominal pain which occurred in 54.9% of patients. Carcinoid syndrome was rare. Only five patients manifested both diarrhea and flushing. 35 patients (12.6%) were diagnosed incidentally without distinct symptoms. Liver was the most common location of distant metastases (86.7%). As for tumor grade, 36.5%, 32.5%, 30.9% of patients had G1, G2 and G3 disease respectively. However, in SEER database, only 5.2% of white, 4.3% of black patients and 9.7% of AP patients had poorly differentiated/undifferentiated disease.

### Comparison of duodenal and jejunal/ileal NENs

Since the tumor location of SI-NENs among different races were significantly different, we then compared the clinical characteristics between duodenal and jejunal/ileal NENs (Table 2). In Chinese patients, tumor size of duodenal NENs (median: 2.0 cm) was significantly smaller than that of jejunal/ileal NENs (median: 3.0 cm). Tumor grades between duodenal NENs and jejunal/ileal NENs were not significantly different. Patients with duodenal NENs were inclined to have T1 (20.1%) and T2 disease (39.6%). Furthermore, duodenal NENs had a lower metastatic rate of lymph nodes compared to that of jejunal/ileal NENs (32.4% vs. 62.7%, *P* < 0.001). Similarly, distant metastasis was less common in duodenal NENs than that in jejunal/ileal NENs but it was not statistically significant. Hence, stage I and stage II were more common in duodenal NENs (*P* < 0.001).

**Table 2:** Comparison of the clinical characteristics between duodenal and jejunal/ileal NENs

	Chinese Patients			White patients			Black patients			Asian/Pacific Islander patients		
	Duodenum	Jejunum/ileum	P	Duodenum	Jejunum/ileum	P	Duodenum	Jejunum/ileum	P	Duodenum	Jejunum/ileum	P
Age, years			0.452			<0.001			<0.001			0.157
Mean	54.7	53.3		63.3	60.7		62.7	57.2		62.5	59.2	
95%CI	52.9–56.6	50.1–56.50		62.6–64.0	60.2–61.1		61.7–63.8	56.1–58.3		60.0–65.0	55.6–62.8	
Sex			0.041			0.914			0.559			0.527
Male	113 (53.3%)	44 (67.7%)		763 (49.5%)	1631 (49.7%)		264 (46.4%)	187 (44.5%)		60 (48.8%)	26 (54.2%)	
Female	99 (46.7%)	21 (32.3%)		778 (50.5%)	1652 (50.3%)		305 (53.6%)	233 (55.5%)		63 (51.2%)	22 (45.8%)	
Tumor size, cm			<0.001			<0.001			<0.001			0.001
Median	2.0	3.0		1.0	1.9		1.0	1.9		1.0	1.5	
Tumor grade^a^	191^b^	58^c^	0.227			-			-			-
Grade 1	74 (38.7%)	17 (29.3%)										
Grade 2	57 (29.8%)	24 (41.4%)										
Grade 3	60 (31.4%)	17 (29.3%)										
Tumor differentiation			-	658^d^	1647^e^	<0.001	223^f^	202^g^	0.149	47^h^	21^i^	0.232
Well differentiated				497 (75.5%)	1268 (77.0%)		171 (76.7%)	169 (83.7%)		38 (80.9%)	14 (66.7%)	
Moderately differentiated				98 (14.9%)	321 (19.5%)		43 (19.3%)	25 (12.4%)		4 (8.5%)	5 (23.8%)	
Poorly or undifferentiated				63 (9.6%)	58 (3.5%)		9 (4.0%)	8 (4.0%)		5 (10.6%)	2 (9.5%)	
T status	169^b^	53^c^	<0.001	890^d^	2559^e^	<0.001	300^f^	339^g^	<0.001	72^h^	38^i^	<0.001
T1	34 (20.1%)	3 (5.7%)		497 (55.8%)	283 (11.1%)		165 (55.0%)	33 (9.7%)		34 (47.2%)	5 (13.2%)	
T2	67 (39.6%)	9 (17.0%)		279 (31.3%)	584 (22.8%)		109 (36.3%)	102 (30.1%)		27 (37.5%)	9 (23.7%)	
T3	56 (33.1%)	35 (66.0%)		62 (7.0%)	1044 (40.8%)		17 (5.7%)	128 (37.8%)		6 (8.3%)	14 (36.8%)	
T4	12 (7.1%)	6 (11.3%)		52 (5.8%)	648 (25.3%)		9 (3.0%)	76 (22.4%)		5 (6.9%)	10 (26.3%)	
N status	188^b^	59^c^	<0.001	1332^d^	3146^e^	<0.001	479^f^	401^g^	<0.001	109^h^	47^i^	<0.001
N0	127 (67.6%)	22 (37.3%)		1154 (86.6%)	930 (29.6%)		421 (87.9%)	142 (35.4%)		94 (86.2%)	15 (31.9%)	
N1	61 (32.4%)	37 (62.7%)		178 (13.4%)	2216 (70.4%)		58 (12.1%)	259 (64.6%)		15 (13.8%)	32 (68.1%)	
M status	212^b^	65^c^	0.090	1308^d^	2672^e^	<0.001	458^f^	353^g^	<0.001	119^h^	48^i^	<0.001
M0	171 (80.7%)	46 (70.8%)		1217 (93.0%)	1908 (71.4%)		434 (94.8%)	267 (75.6%)		112 (94.1%)	36 (75.0%)	
M1	41 (19.3%)	19 (29.2%)		91 (7.0%)	764 (28.6%)		24 (5.2%)	86 (24.4%)		7 (5.9%)	12 (25.0%)	
Tumor stage	183^b^	58^c^	<0.001	817^d^	2548^e^	<0.001	273^f^	330^g^	<0.001	76^h^	47^i^	<0.001
I	30 (16.4%)	2 (3.4%)		407 (49.8%)	132 (5.2%)		134 (49.1%)	16 (4.8%)		34 (44.7%)	5 (10.6%)	
II	75 (41.0%)	13 (22.4%)		190 (23.3%)	305 (12.0%)		75 (27.5%)	53 (16.1%)		20 (26.3%)	7 (14.9%)	
III	37 (20.2%)	24 (41.4%)		129 (15.8%)	1347 (52.9%)		40 (14.7%)	175 (53.0%)		15 (19.7%)	23 (48.9%)	
IV	41 (22.4%)	19 (32.8%)		91 (11.1%)	764 (30.0%)		24 (8.8%)	86 (26.1%)		7 (9.2%)	12 (25.5%)	

^a^Tumor grade was based on WHO 2010 classification criteria (grade 1, 2 and 3) in Chinese patients. ^b,c^The number available in Chinese SI-NENs patients with 212 patients of duodenal NENs and 65 patients of jejunal/ileal NENs in total. ^d,e^The number available in SEER database with 1541 white patients of duodenal NENs and 3283 white patients of jejunal/ileal NENs in total. ^f,g^The number available in SEER database with 569 black patients of duodenal NENs and 420 black patients of jejunal/ileal NENs in total. ^h, i^The number available in SEER database with 123 Asian/Pacific Islander patients of duodenal NENs and 48 Asian/Pacific Islander patients of jejunal/ileal NENs

In patients from SEER database, similar results were found (Table 2). Tumor size was also significantly smaller and T1, T2, stage I and stage II were also more common in patients with duodenal NENs than jejunal/ileal NENs. Both metastatic rate of lymph nodes and distant location were significantly lower in duodenal NENs compared with jejunal/ileal NENs. The mean age of patients with duodenal NENs were older than patients with jejunal/ileal NENs both in white and black patients but not in AP patients. Additionally, in white patients but not black and AP patients, poorly differentiated tumor was more common in duodenal NENs compared with jejunal/ileal NENs (9.6% vs. 3.5%).

### Comparison of ampullary and non-ampullary duodenal NENs

We further compared the clinicopathological features of ampullary NENs and non-ampullary NENs (Table 3). In Chinese patients, the most common symptoms of patients with ampullary NENs were abdominal pain, followed by jaundice. While in non-ampullary duodenal NENs, jaundice was less common. G3 disease (47.6%) was more common and tumor size (median, 2.5 cm) was larger in ampullary NENs compared with non-ampullary duodenal NENs. Patients with ampullary NENs tended to have more T3 and T4 disease. However, ampullary NENs did not show more metastases to lymph nodes (N1) and distant location (M1) compared with non-ampullary duodenal NENs.

**Table 3:** Comparison of the clinical characteristics between ampullary and non-ampullary duodenal NENs

Characteristics	Chinese patients			White patients			Black patients		
	Ampullary NENs	Non-ampullary duodenal NENs	P	Ampullary NENs	Non-ampullary duodenal NENs	P	Ampullary NENs	Non-ampullary duodenal NENs	P
Age, years			0.961			0.003			0.028
Mean (95%CI)	54.9 (51.9–58.0)	54.8 (52.3–57.4)		59.7 (57.1–62.3)	63.6 (62.9–64.3)		57.1 (52.5–61.7)	63.0 (61.9–64.0)	
Range	25–81	17–80		31–87	13–97		34–80	21–94	
Sex	72^a^	122^b^	0.337	117^c^	1424^d^	0.691	24^e^	545^f^	0.435
Male	35 (48.6%)	68 (55.7%)		60 (51.3%)	703 (49.4%)		13 (54.2%)	251 (46.1%)	
Female	37 (51.4%)	54 (44.3%)		57 (48.7%)	721 (50.6%)		11 (45.8%)	294 (53.9%)	
Tumor size, cm	61^a^	93^b^	0.010	86^c^	851^d^	<0.001	21^e^	312^f^	<0.001
Median	2.5	1.6		1.8	0.9		2.2	1.0	
Presenting Symptoms	72^a^	122^b^	-			-			-
Abdominal pain	34 (47.2%)	63 (51.6%)							
Jaundice	26 (36.1%)	7 (5.7%)	<0.001^g^						
Nausea/vomiting	8 (11.1%)	17 (13.9%)							
Bloating	5 (6.9%)	23 (18.9%)							
GI bleeding	8 (11.1%)	16 (13.1%)							
Diarrhea	3 (4.2%)	11 (9.0%)							
Intestinal obstruction	4 (5.6%)	3 (2.5%)							
Flushing	2 (2.8%)	2 (1.6%)							
Incidental diagnosis	72^a^	122^b^	0.386						
Yes	8 (11.1%)	19 (15.6%)							
No (symptomatic)	64 (88.9%)	103 (84.4%)							
Tumor grade	63^a^	112^b^	0.003			-			-
Grade 1	18 (28.6%)	48 (42.9%)							
Grade 2	15 (23.8%)	39 (34.8%)							
Grade 3	30 (47.6%)	25 (22.3%)				<0.001			0.177
Tumor differentiation			-	63^c^	595^d^		15^e^	209^f^	
Well differentiated				28 (44.4%)	469 (78.8%)		10 (66.7%)	161 (77.0%)	
Moderately differentiated				12 (19.0%)	86 (14.5%)		3 (20.0%)	40 (19.1%)	
Poorly or undifferentiated				23 (36.5%)	40 (6.7%)		2 (13.3%)	8 (3.8%)	
T status	60^a^	95^b^	<0.001	78^c^	812^d^	<0.001	19^e^	281^f^	0.002
T1	4 (6.7%)	29 (30.5%)		15 (19.2%)	482 (59.4%)		3 (15.8%)	162 (57.7%)	
T2	20 (33.3%)	39 (41.1%)		39 (50.0%)	240 (29.6%)		13 (68.4%)	96 (34.2%)	
T3	30 (50.0%)	21 (22.1%)		9 (11.5%)	53 (6.5%)		2 (10.5%)	15 (5.3%)	
T4	6 (10.0%)	6 (6.3%)		15 (19.2%)	37 (4.6%)		1 (5.3%)	8 (2.8%)	
N status	70^a^	102^b^	0.535	97^c^	1235^d^	<0.001	22^e^	457^f^	<0.001
N0	47 (67.1%)	73 (71.6%)		51 (52.6%)	1103 (89.3%)		13 (59.1%)	408 (89.3%)	
N1	23 (32.9%)	29 (28.4%)		46 (47.4%)	132 (10.7%)		9 (40.9%)	49 (10.7%)	
M status	72^a^	122^b^	0.071	110^c^	1198^d^	<0.001	22^e^	436^f^	0.001
M0	64 (88.9%)	96 (78.7%)		87 (79.1%)	1130 (94.3%)		17 (77.3%)	417 (95.6%)	
M1	8 (11.1%)	26 (21.3%)		23 (20.9%)	68 (5.7%)		5 (22.7%)	19 (4.4%)	
Tumor stage	64^a^	102^b^	<0.001	90^c^	727^d^	<0.001	20^e^	253^f^	0.005
I	3 (4.7%)	26 (25.5%)		8 (8.9%)	399 (54.9%)		3 (15.0%)	131 (51.8%)	
II	30 (46.9%)	39 (38.2%)		16 (17.8%)	174 (23.9%)		8 (40.0%)	67 (26.5%)	
III	23 (35.9%)	11 (10.8%)		43 (47.8%)	86 (11.8%)		4 (20.0%)	36 (14.2%)	
IV	8 (12.5%)	26 (25.5%)		23 (25.6%)	68 (9.4%)		5 (25.0%)	19 (7.5%)	

*GI* gastrointestinal. ^a, b^The number available in 72 patients with ampullary NENs and 122 patients with non-ampullary duodenal NENs; ^c,d^The number available in 117 patients with ampullary NENs and 1424 patients with duodenal NENs; ^e,f^The number available in 117 patients with ampullary NENs and 1424 patients with duodenal NENs; ^g^Jaundice was significantly more common in patients with ampullary NENs

Due to the small sample size of ampullary NENs in AP patients, the comparison of ampullary and non-ampullary duodenal NENs was not performed. In white and black patients from SEER database, the mean age of patients with ampullary NENs was younger than that of non-ampullary duodenal NENs. Tumor size of ampullary NENs was also significantly larger. Ampullary NENs tended to be more aggressive that T3, T4, N1 and M1 disease were more common than that in non-ampullary duodenal NENs. Therefore, stage III and IV disease were more common in ampullary NENs. Additionally, in white patients, poorly differentiated NENs consisted of a larger proportion (36.5%) in ampullary NENs than that in non-ampullary duodenal NENs (6.7%).

### Tumor grade and survival

Tumor grade according to WHO 2010 classification was available in Chinese patients but not in patients from SEER database. Hence, the prognostic validity of WHO grading classification was analyzed only in Chinese patients. 207 patients with survival information including 160 patients (77.3%) with duodenal NENs and 47 patients (22.2%) with jejunal/ileal NENs were retrieved. The mean follow-up time was 24.3 months (95%CI, 20.9 to 27.6 months). The 5-year overall survival (OS) rates of G1, G2 and G3 were 91.7% (95%CI, 83.9% to 99.5%), 76.6% (95%CI, 60.5% to 92.7%) and 32.9% (95%CI, 18.4% to 47.4%) respectively. The survival rate of patients with G3 was significantly worse than that of patients with G1 and G2 (G3 vs. G1, *P* < 0.001; G3 vs. G2, *P* < 0.001). However, survival rate of patients with G1 and G2 was not significantly different (G2 vs. G1, *P* = 0.132). Multivariate analysis adjusting for age, sex, tumor location, tumor stage revealed similar results (G2 vs. G1, HR 1.89, 95%CI, 0.57 to 6.28, *P* = 0.301; G3 vs. G1, HR, 11.48, 95%CI, 3.91 to 33.69, *P* < 0.001).

**Fig. 1:**
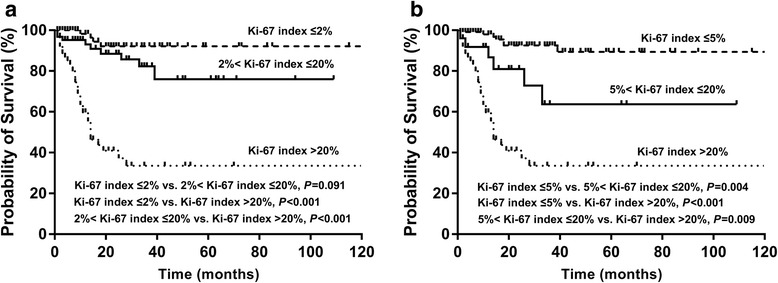
Kaplan-Meier analysis of Chinese patients with SI-NENs according to Ki-67 index classification. **a** With Ki-67 index of 2% as threshold, survival rate of G1 and G2 was not significantly different. **b** With Ki-67 index of 5% and 20% as threshold between grade 1/2 and grade 2/3 respectively, survival rate among different grades were significantly different

Previous studies suggested Ki-67 index of 5% as the threshold between G1 and G2 disease in pancreatic and midgut NENs [11, 12]. In our study, we also investigated whether 2% or 5% was the better threshold between G1 and G2. With Ki-67 index of 2% as threshold, Kaplan-Meier analysis with log-rank test did not show significantly different result between G1 and G2 (*P* = 0.091; Fig. 1a). With Ki-67 index of 5% as threshold, G2 had significantly worse survival compared with G1 (Fig. 1b; *P* = 0.004). Table 4 showed the results of analyses of potential factors of OS. Factors which were statistically significant associated with OS in univariate analyses were included in the multivariate analyses. Multivariate analysis showed that, G2 was a significant factor indicating poorer survival when Ki-67 index of 5% but not 2% as threshold (Table 4). In addition, stage IV was also an independently prognostic factor indicating worse survival (Table 4).

**Table 4:** Univariate and multivariate analysis of overall survival in patients with SI-NENs: comparison of Ki-67 index 2 and 5% as threshold

Factor (N)	Univariate analysis		Multivariate analysis			
			2% as threshold		5% as threshold	
	HR (95% CI)	P	HR (95% CI)	P	HR (95% CI)	P
Age (207)	1.02 (0.99–1.04)	0.204				
Sex		0.502				
Male (121)	1.00					
Female (86)	0.82 (0.45–1.48)					
Location of primary tumor						
Ampulla (54)	1.00					
Non-ampulla duodenum (106)	0.69 (0.35–1.34)	0.272				
Jejunum/ileum (47)	0.62 (0.29–1.34)	0.223				
Tumor stage		0.002		0.022		0.033
Stage I/II (95)	1.00		1.00		1.00	
Stage III (44)	2.43 (1.01–5.87)	0.048	1.89 (0.78–4.58)	0.158	1.90 (0.79–4.61)	0.155
Stage IV (48)	4.68 (2.13–10.27)	<0.001	3.44 (1.55–7.62)	0.002	3.29 (1.48–7.31)	0.003
Unknown (20)	2.90 (1.03–8.15)	0.044	2.26 (0.80–6.37)	0.124	2.17 (0.77–6.13)	0.144
Ki-67 index		<0.001		<0.001		
≤ 2% (80)	1.00		1.00			
3%–20% (63)	2.67 (0.82–8.68)	0.102	2.18 (0.66–7.15)	0.199		
> 20% (64)	14.73 (5.20–41.74)	<0.001	12.21 (4.26–34.98)	<0.001		
Ki-67 index		<0.001				<0.001
≤ 5% (118)	1.00				1.00	
6%–20% (25)	4.29 (1.44–12.76)	0.009			3.28 (1.08–9.98)	0.036
> 20% (64)	12.98 (5.70–29.56)	<0.001			11.31 (4.92–26.01)	<0.001

## Discussion

In this study, we collected a series of patients with SI-NENs from eleven Chinese hospitals which was the largest series from Asia. We found that the clinicopathological characteristics of SI-NENs in different races were different. One of the most distinct characteristics we found was that the rate of duodenal NENs was significantly higher in Chinese patients than that in white and black patients from SEER database. This situation was also found in AP patients with SI-NENs from SEER database. Therefore, the most potential cause might be genetic differences among different races. The most common manifestation was abdominal pain in Chinese patients while carcinoid syndrome with flushing and diarrhea was much rarer. This might because most of Chinese patients having NENs located in duodenum while carcinoid syndrome mostly occurred in patients with advanced NENs of distal small intestine [16]. We also found that G3 disease (mostly poor differentiation) was more common in Chinese SI-NENs patients while less than 10% of white, black and AP patients from SEER database had poorly differentiated tumor. Patients with ampullary NENs in the Chinese series were more common than that from SEER database. Furthermore, ampullary NENs had more G3 disease compared with other location. This might partly explain the high frequency of G3 disease in Chinese patients.

The clinicopathological characteristics of duodenal NENs and jejunal/ileal NENs were also different. In the Chinese cohort, we found localized disease was more common in duodenal NENs. Tumor size was smaller, while T1, T2, N0 and M0 disease were more common in duodenal NENs. These findings were confirmed by the data from SEER database. The heterogeneities between duodenal and jejunal/ileal NENs indicated that NENs in these two locations might have different biological behaviors which should be managed differently [17, 18]. Since duodenal NENs were quite different from jejunal/ileal NENs, different distribution of tumor might be one of the reasons causing different clinicopathological characteristics of SI-NENs among Chinese patients and white and black patients from SEER database.

In addition, we found ampullary NENs and non-ampullary duodenal NENs shared different characteristics. Since the growth of tumor originating from ampulla or major duodenal papilla might cause obstruction of bile duct, jaundice was one of the most common symptoms of ampullary NENs but not non-ampullary duodenal NENs. Tumor size was significantly larger in ampullary NENs. This might due to the more aggressive nature of ampullary NENs since G3 disease or poorly differentiated tumor were more common in ampullary NENs. In patients from SEER database, metastases of lymph nodes and distant location were more common in ampullary NENs than that in non-ampullary duodenal NENs. However, in Chinese cohort, patients with ampullary NENs did not have more lymphatic or distant metastases than non-ampullary duodenal NENs. Furthermore, ampullary NENs was rare in Asian/Pacific Islander. The potential reason might be that in Chinese cohort, more NENs from periampullary duodenum were included as ampullary NENs in which localized lesion might be more common. It was sometimes challenging to differentiate whether the large periampullary neoplasms were originating from ampulla of Vater or not.

WHO grading classification proposed in 2010 is widely used in evaluating prognosis of patients with NENs [8, 11, 19, 20]. In our study, we also confirmed the value of this classification in Chinese SI-NENs patients. G3 disease had significantly worse survival compared with G1 and G2 disease. Ki-67 index threshold to differentiate G1 and G2 remained controversial. A study of midgut NENs revealed Ki-67 index threshold of 2% between G1 and G2 was enough to distinguish patients with different disease specific survival [8]. However, a study of 274 pancreatic NENs indicated that Ki-67 threshold of 5% rather than 2% was an optimal threshold between G1 and G2 disease [11]. This result was confirmed by another study which suggested the threshold between G1 and G2 should be revised from 2% to 5% both in pancreatic and midgut NENs [12]. Another study of duodenal NENs revealed that disease-specific survival of G1 and G2 (with Ki-67 index of 2% as threshold) was significantly different only in univariate analysis but not in multivariate analysis after adjusting for age and stage [21]. In our study, we found Ki-67 index of 5% might be better than 2% to differentiate two groups with significantly different outcome. The result was further verified by multivariate analysis. Therefore, we considered Ki-67 index of 2% as threshold between G1 and G2 was also questionable in Chinese patients with SI-NENs.

Limitations still exist in our study. The first limitation is the retrospective nature of our study. But we include more than 270 Chinese patients with SI-NENs which is the largest series from Asia, and we also specify a large sample of patients with SI-NENs from SEER database. The second limitation is that information of Ki-67 index is not available in SEER database so that the result about the WHO grading classification in this study cannot be confirmed by patients from SEER database. Hence, more studies are still required to verify our result. The third limitation is that we compare a Chinese multicenter cohort with a population-based database and there may be a chance of referral bias in the Chinese cohort. To make the Chinese cohort more representative and the referral bias as low as possible, we retrieved data from more than ten representative general hospitals and cancer centers located in different geographical regions of China.

## Conclusion

The clinicopathological characteristics of SI-NENs are quite different among different races which may due to different location of tumor. Duodenum is the predominant location of SI-NENs in Chinese patients and AP patients but not in white patients. Duodenal NENs and jejunal/ileal NENs, ampullary NENs and non-ampullary duodenal NENs also share different clinicopathological features. G3 and stage IV disease are independent factors indicating worse survival. Ki-67 index of 5% may be a better threshold between G1 and G2 in patients with SI-NENs.

## Abbreviations

AJCC: American Joint Committee on Cancer
AP: Asian/Pacific Islander
CI: Confidence interval
ENETS: European Neuroendocrine Tumor Society
HR: Hazard ratio
MEN-1: Multiple endocrine neoplasia type 1
OS: Overall survival
SEER: Surveillance, Epidemiology, and End Results
SI-NENs: Small intestinal neuroendocrine neoplasms
WHO: World Health Organization
